# Change of the aortic elasticity in rheumatoid arthritis: Relationship to associated cardiovascular risk factors

**DOI:** 10.4103/0975-3583.70901

**Published:** 2010

**Authors:** Hamdy Sliem, Gamela Nasr

**Affiliations:** *Department of Internal Medicine, Suez Canal University, Egypt*; 1 *Department of Cardiology, Suez Canal University, Egypt*

**Keywords:** Aortic elasticity, rheumatoid arthritis, risky factors

## Abstract

**Introduction:**

Rheumatoid arthritis (RA) is a chronic, systemic, inflammatory disease, which is associated with an excess of cardiovascular events. A decrease in the compliance of the arterial system, termed arterial stiffness, results in increased cardiac workload. Primary prevention of cardiovascular disease (CVD) is a priority for modern medicine. Therefore, further studies are required to explore the mechanisms through which CVD increases in RA.

**Patients and Methods:**

This case–control study was performed to detect possible change of aortic elasticity in patients with RA, and to estimate the impact of different cardiovascular and atherogenic risk factors on the severity of arterial stiffness. Sixty-three consecutive adults with RA were enrolled for the study (case group). Forty-one healthy adults matched for age and gender were considered as a control group. All were subjected to assessment of aortic stiffness index and various cardiovascular risk factors. Patients with rheumatoid disease (case group) were divided by their aortic stiffness index status to two groups (A and B, with and without aortic stiffness, respectively).

**Results:**

Aortic stiffness was present in 31.7% of the RA patients. Age of the patients, duration of RA, smoking index, waist circumference, triglycerides levels, and CRP were significantly higher in patients with aortic stiffness.

**Conclusion:**

RA is associated with decreased elasticity of the aorta in both genders, and such changes seem to be higher in the presence of visceral obesity, smoking, high triglycerides, and extraarticular disease severity.

## INTRODUCTION

Rheumatoid arthritis (RA) is a chronic, systemic, inflammatory disease.[1] It is associated with an increased mortality compared with the general population, mainly due to an excess of deaths from cardiovascular disease (CVD).[2–4] Disability and severe extraarticular disease manifestations are predictors of poor survival in patients with RA.[5]

Hyperglycemia, hypertension, dyslipidemia, obesity, smoking, and aging are associated with high risk of CVD as well as other clinical conditions.[6] The mechanisms through which cardiovascular risk is increased are partially understood. A decrease in the compliance of the central arterial system, termed arterial stiffness, results in increased cardiac workload.[7] Some studies had revealed that aortic stiffness, wave-reflection intensity, and endothelial function are independent predictors of cardiovascular risk in various patient groups and may also directly accelerate the atherosclerotic process.[8–10]

A number of factors are believed to be responsible for arterial stiffness, which include decreased elastin and increased collagen in the arterial wall, and abnormal endothelial regulation of arterial smooth muscle tone.[6]

Primary prevention of CVD is a priority for modern medicine.[1,11] Therefore, there is the need for more studies to explore the mechanisms through which CVD increases in RA. This study was undertaken in order to study the distensibility of elastic aorta in patients with RA, and to estimate the impact of different cardiovascular and atherogenic risk factors on the severity of arterial stiffness.

## PATIENTS AND METHODS

### Patient selection

A case–control study was performed. Sixty-three consecutive adults with RA were enrolled for the study (case group). All were recruited from the outpatient rheumatology, cardiology, and internal medicine clinics of Suez Canal University Hospital from May 2007 to February 2008. Forty-one healthy adults matched for age and gender were considered as a control group.

The diagnosis of RA was defined according to the proposed American College of Rheumatology criteria: 1. Morning stiffness, 2. Arthritis of three or more joint areas, 3. Arthritis of hand joints, 4. Symmetric arthritis, 5. Rheumatoid nodules, 6. Serum rheumatoid factor, and 7. Radiographic changes. A patient was diagnosed as RA if he/she had satisfied at least four of these seven criteria. Criteria 1 through 4 must have been present for at least 6 weeks.[12] Patients with chronic liver, chronic kidney, personal or family history of overt CVDs, other rheumatic or collagen diseases were excluded from the study.

All groups were subjected to full medical history, clinical examination including blood pressure (BP), body mass index (BMI), systemic examination, biochemical radiological, and echocardiographic studies. Smoking index was calculated as the product of the number of cigarettes smoked per day and the number of years for which they had been smoked.[13] BMI was calculated as weight/height^2^ (kg/m^2^) and was used as an estimate of overall adiposity. Waist circumference, a validated estimate of visceral adiposity, was measured to the nearest 0.5 cm. Central obesity was defined as (circumference > 102 cm in males and >88 cm in females).[14] BP was measured from the brachial artery with a manual cuff, and mean arterial pressure (MAP) was calculated using the formula MAP = DBP + (SBP – DBP)/3, where SBP is systolic blood pressure and DBP is diastolic blood pressure.[15] CRP assay via chemiluminescence using IMMULITE/ IMMULITE 1000 reagent. Cholesterol, triglycerides, highdensity lipoproteins, blood glucose, and rheumatoid factor via automated HITACHI 92 autoanalyzer and automated OBAS INTEGRA 400 autoanalyzer.

Aortic stiffness index (β) as a characteristic of aortic elasticity was evaluated from ascending aortic diameter. Aortic stiffness index (β) was evaluated by means of transthoracic echocardiography by use of the formula: β = ln(SBP/DBP)/(∆D/DD), where SBP and DBP are the systolic and diastolic BPs, DD is the diastolic aortic diameter, ∆D is the pulsatile change in aortic diameter (systolic diameter minus diastolic diameter) and ‘ln’ is the natural logarithm).[8,16]

Patients with rheumatoid disease (case group) were divided by their aortic stiffness index status at baseline (β above and below median of 3.0) to two groups (A and B, with and without aortic stiffness, respectively). The median was based on the overall control group at baseline.

### Ethical consideration

Informed consent was obtained from all the adults. The aim and the value of the work were explained in a simplified manner for them. There was no harm inflicted on them. On the contrary, all had benefits of the follow-up and the final results of the study. The study was approved by the Ethics Committee of Faculty of Medicine, Suez Canal University.

### Statistical analysis

According to the type of data, the student’s unpaired *t*-test and Chi-square test were used for statistical comparisons of two groups. Descriptive statistics were done including mean, standard deviation, mode and median for quantitative variables, and frequency and percent for qualitative variables. The analysis was carried out by a computer program (SPSS Ver. 11). *P* value was set at <0.05 for statistically significant results and <0.0001 for highly significant results.

## RESULTS

Baseline demographic and clinical characteristics of 63 (33 males and 30 females) RA patients, mean age 44.1 years, with and without aortic stiffness versus 41 control (22 males and 19 females), mean age 45.8 years are shown in Table 1. Of them, 31.7% of the patients had aortic stiffness (Group A). No significant differences were observed between both patient and control groups in terms of age, gender distribution, BMI, and BP. Except waist circumference, all mentioned clinical parameters were nearly similar in both male and female patients. Mean of age, smoking index, duration of RA, percent of extraarticular manifestations were significantly higher in Group A than that of Group B. All RA patients were treated by nonsteroidal antiinflammatory drugs. Methotrexate was taken by 17 cases (27%), 5 in Group A (25%) and 12 in Group B (28%).

**Table 1:** Demographic and clinical studies of both case and control groups

Variables	Control group, *N* = 41	Patients with RA (case), *N*=63			*P* value		
		All, N = 63	Group A, *N* = 20 (31.7%)	Group B, *N* = 43 (68.3%)	*P*	P*	P**
Age (years)	45.8 ± 7.9	44.1 ± 6.2	52.9 ± 7.1	39.9 ± 5.9	<0.05	<0.05	n.s
Gender							
Male (%)	*N* = 22 (54)	*N* = 33 (52)	*N* = 11 (55)	*N* = 22 (51)	n.s	n.s	n.s
Female (%)	*N* = 19 (46)	*N* = 30 (48)	*N* = 9 (45)	*N* = 21 (49)	n.s	n.s	n.s
RA duration (years)	–	9 ± 2.3	13 ± 2.9	7 ± 3.1	–	<0.05	–
Smoking index	43 ± 5.4	36 ± 6.3	62 ± 5.1	31 ± 7.8	<0.01	<0.05	n.s
SBP (mmHg)	121.9 ± 7.2	122.2 ± 9.2	124.9 ± 7.9	120.1 ± 5.3	n.s	n.s	n.s
DBP (mmHg)	74.4 ± 3.1	76.3 ± 2.1	75.9 ± 2.5	73.9 ± 1.6	n.s	n.s	n.s
Mean BP (mmHg)	74.4 ± 3.1	76.3 ± 2.1	75.9 ± 2.5	73.9 ± 1.6	n.s	n.s	n.s
BMI %	23.6 ± 5.7	25.4 ± 5.2	26.5 ± 5.2	24.4 ± 5.1	n.s	n.s	n.s
W. circum (cm)	100.3 ± 16.3	103.2 ± 17.2	112.4 ± 11.6	95.5 ± 18.2	<0.01	< 0.05	n.s
Extraarticular m. (%)	–	*N* = 21(33)	*N* = 12 (60)	*N* = 9 (21)	–	<0.05	–

Group A = patients with aortic stiffness; Group B = patients without aortic stiffness; RA = rheumatoid arthritis; BMI = body mass index; SBP = systolic blood pressure; DBP = diastolic blood pressure; Mean BP = mean blood pressure; W. circum = waist circumference; N = number of cases; n.s = non significant; Extraarticular m. = Extraarticular manifestations. P = comparison between Group A and control.

P* = comparison between Groups A and B.

P** = comparison between all patients and control group

Baseline biochemical characteristics are presented in Table 2. Mean values of triglycerides and C-reactive protein (CRP) were significantly higher in Group A than that of Group B. On the other hand, mean values of low- and high-density lipoproteins, fasting plasma glucose, and rheumatoid factor percents were similar in both Group A and Group B. Similarly, no differences were found with overall patients and control variables.

**Table 2:** Biochemical studies of both case and control groups

Variables	Control group, *N* = 41	Patients with RA (case), *N*=63			*P* value		
		All, *N* = 63	Group A, *N* = 20 (31.7%)	Group B, *N* = 43 (68.3%)	*P*	P*	P**
HDL mg%	48.1±5.2	44.1 ± 3.9	41.9 ± 4.5	45.1 ± 4.2	n.s	n.s	n.s
LDL mg%	112.9 ± 9.3	117.7 ± 2.4	127.2 ± 16.7	121.3 ± 15.2	n.s	n.s	n.s
Triglycerides mg%	146.8 ± 15.6	181.4 ± 14.5	197.5 ± 14.2	187.5 ± 11.9	<0.01	<0.05	<0.05
FPG mg%	83.1 ± 7.2	91.2 ± 7.8	114.8 ± 14.6	101.7 ± 19.9	n.s	n.s	n.s
CRP	12.3 ± 1.9	22.7 ± 6.2	31.2 ± 4.1	17.2 ± 3.2	<0.01	<0.05	<0.05
RF (positive)	–	*N* = 48 (76%)	*N* = 15 (75%)	*N* = 33 (77%)	–	n.s	–

Group A = patients with aortic stiffness; Group B = patients without aortic stiffness; LDL = low density lipoprotein; HDL = high density lipoprotein, N = number of cases; n.s = non significant; CRP= C reactive protein; FPS = fasting plasma glucose; RF = rheumatoid factor. P = comparison between Group A and control.

P* = comparison between Groups A and B.

P** = comparison between all patients and control group

**Figure 1:**
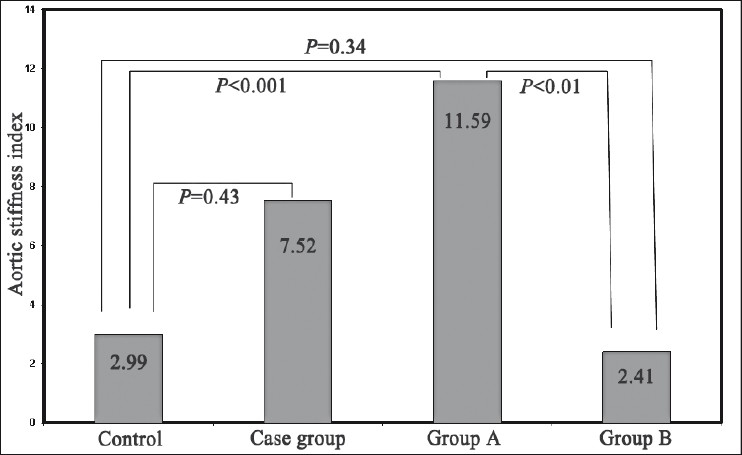
Group A: RA patients with aortic stiffness Group B: RA patients without aortic stiffness. Mean values of aortic stiffness index in case and control groups

Regarding aortic stiffness index, significant difference was observed between all patients’ cases and control group. In addition, highly significant difference was observed between Group A and both Group B and control [Figure 1].

## DISCUSSION

RA is a systemic disease associated with increased mortality, mostly due to an excess of CVD.[17] In recent years, great attention has been paid to the role of changes in the stiffness of the large arteries, such as the aorta and its major branches, in the development of CVDs.[18–20]

In the general population, the elasticity of the aorta decreases more rapidly in men than women.[6,9] In contrary, other studies reported an increased stiffness in women, but not in men in patients with RA.[20,21] However, this study confirmed the presence of an increased stiffness of the aorta associated with RA in both genders. The male and female distribution was nearly similar in both aortic stiffness and non-stiffness rheumatoid patients.

It is known that different arterial segments respond differently to aging. Aorta, an elastic artery, loses its compliance with advancing age, whereas compliance in the peripheral, predominantly muscular, arteries appears less closely related to age.[22] In current data, age was an independent predictor of aortic stiffness.

Duration of rheumatoid seems to have positive reflection on the presence of aortic stiffness. Mean disease duration was significantly higher in RA patients with aortic stiffness than those without. As detected in the current study, 75% of patients with aortic stiffness had disease duration more than 10 years. This could be explained by the fact that inflammatory cytokines play an important role in the pathogenesis of RA and contribute to mediate not only local acute events but also the systemic response.[23] Therefore, one can speculate that chronic cytokine release may lead to the deposition of cellular and lipid debris, producing the characteristic arterial wall changes and dysfunctional vasculature.

Tobacco smoking was further categorized by us after calculation of smoking index. Smoking is regarded to be a powerful predictor of CVD in the general population. Smoking has also been reported to be a predictor of the RA disease itself.[24] Thus, tempting to believe that smoking would be the cause of an increased CVD in RA patients. Its’ burden on the decrease of aortic elasticity was reported. Our results are in agreement with other studies.[2,25]

Previous studies have shown that visceral fat in young healthy individuals and older adults is associated with increased central arterial stiffness.[26] This study confirms this result. In agreement with a recent study by Vizzardi *et al*.[10] we observed that mean of abdominal adiposity as measured by waist circumference was strongly and adversely higher in rheumatoid patients with aortic stiffness, whereas BMI as a measure of general adiposity was not. These results suggest that the rheumatoid–arterial stiffness relationship may be mediated in part through increasing central obesity. In general, adipocytes, in particular from visceral abdominal regions, produce several bioactive peptides which in turn impact on vascular structure and function.[27]

Van Doornum *et al*, reported that stiffness of the aorta tended to be higher in patients with recently diagnosed severe extraarticular manifestations, and there was a significant association between disability and aortic stiffness.[28] In our study, the presence or absence of extraarticular manifestations showed significant reflection on the state of aortic elasticity. This is in agreement with other studies. The scientists from three different Swedish universities examined the distensibility and the diameter of the abdominal aorta and the common carotid artery by echo-tracking ultrasonography in 101 patients with RA. The results were compared with healthy individuals from a corresponding age group. In the RA cohort, patients with extraarticular manifestations tended to have greater stiffness of the aorta. Also disability, as indicated by a Higher Health Assessment Questionnaire Score, was associated with increased aortic stiffness.[18,29] This indicates that other several disease severity may have an impact on arterial wall abnormalities in patients with RA.

However, increased distending pressure tends to reduce the elasticity of a given arterial segment through the recruitment of collagen fibers. In addition, several studies have shown that arterial stiffness is increased in individuals with diabetes.[9,25,30] Nevertheless, in our study, most of the patients were nearly normotensive and euglycemic, such the association was not held.

We should point out that, in current data the total lipids had insignificant impact on aortic stiffness. When the components of the lipids were considered separately, aortic stiffness showed direct associations only with triglycerides. Similar conclusion was reported in a previous study of aortic stiffness in metabolic syndrome.[16]

CRP levels predict outcome in healthy individuals and patients with atherosclerosis. The increased CRP levels predict cardiovascular morbidity in early inflammatory polyarthritis. Increased CRP and erythrocyte sedimentation rate (ESR) are associated with greater thickness of the carotid artery intima-media in RA and also in healthy subjects.[31] Others have found CRP levels to be associated with aortic pulse wave velocity in healthy individuals and in patients with systemic vasculitis.[32] Some authors did not find any association between current CRP levels and aortic stiffness.[33] However, in this study current CRP levels were higher in rheumatoid patients with aortic stiffness than those without, suggesting that inflammation may be involved in arterial stiffening. Anti-inflammatory strategies may, therefore, be of benefit in reducing arterial stiffness and thus CVD.[34] In addition, Kaisa *et al*. suggested that effective control of inflammation may reduce cardiovascular risk in patients with RA as it improves arterial stiffness and endothelial function, both of which are established surrogate measures of cardiovascular risk. Moreover, RA may serve as a useful model to investigate the effects of other immunomodulatory agents on arterial stiffness and endothelial function.[35]

## CONCLUSION

RA is associated with decreased elasticity of the aorta in both genders, and such changes seem to be higher in patients with extraarticular disease severity. Arterial stiffness may act as a marker for the development of future CVD in RA. Measurement of arterial stiffness will become an increasingly important part of the process of risk assessment, and may possibly improve the monitoring of therapy. Various risk factors have impact on the increased arterial stiffness parameters. Apart from the dominant effect of aging, they include smoking, visceral obesity, triglyceride, CRP, hypertension, and hyperglycemia. Parallel to treatment of rheumatoid, appropriate and watchful dealing with all associated risk factors should be considered. The main limitation of the study was the sample size. Additional studies will be needed to assess the effect of anti-inflammatory and immunomodulatory agents to decrease arterial stiffness and the impact of the rheumatoid disease activity on the severity of arterial stiffness.
